# Robust Confidence Intervals for PM_2.5_ Concentration Measurements in the Ecuadorian Park La Carolina

**DOI:** 10.3390/s20030654

**Published:** 2020-01-24

**Authors:** Wilmar Hernandez, Alfredo Mendez, Rasa Zalakeviciute, Angela Maria Diaz-Marquez

**Affiliations:** 1 Facultad de Ingeniería y Ciencias Aplicadas, Universidad de Las Américas, Quito 170125, Ecuador; 2 Departamento de Matemática Aplicada a las Tecnologías de la Información y las Comunicaciones, ETS de Ingeniería y Sistemas de Telecomunicación, Universidad Politécnica de Madrid, 28031 Madrid, Spain; alfredo.mendez@upm.es; 3 Grupo de Biodiversidad, Medio Ambiente y Salud (BIOMAS), Universidad de Las Américas, Quito 170125, Ecuador; rasa.zalakeviciute@udla.edu.ec; 4 Grupo Dinámicas + Lugar, Medio y Sociedad (D + LMS), Universidad de Las Américas, Quito 170125, Ecuador; angela.diaz@udla.edu.ec

**Keywords:** PM_2.5_ concentration measurements, robust location estimation, robust scale estimation, robust confidence intervals, air pollution classification

## Abstract

In this article, robust confidence intervals for PM_2.5_ (particles with size less than or equal to 2.5 μm) concentration measurements performed in La Carolina Park, Quito, Ecuador, have been built. Different techniques have been applied for the construction of the confidence intervals, and routes around the park and through the middle of it have been used to build the confidence intervals and classify this urban park in accordance with categories established by the Quito air quality index. These intervals have been based on the following estimators: the mean and standard deviation, median and median absolute deviation, median and semi interquartile range, a-trimmed mean and Winsorized standard error of order a, location and scale estimators based on the Andrew’s wave, biweight location and scale estimators, and estimators based on the bootstrap-t method. The results of the classification of the park and its surrounding streets showed that, in terms of air pollution by PM_2.5_, the park is not at caution levels. The results of the classification of the routes that were followed through the park and its surrounding streets showed that, in terms of air pollution by PM_2.5_, these routes are at either desirable, acceptable or caution levels. Therefore, this urban park is actually removing or attenuating unwanted PM_2.5_ concentration measurements.

## 1. Introduction

Particulate matter (PM) is a mixture of particles of different compositions, sizes, and origins, which for different reasons are in the air [1]. According to [1], the range of values of the aerodynamic diameter of these particles is from less than 100 nm up to a few micrometers. In accordance with [2], these particles can be classified according to their size into coarse particles (size from 2.5 μm up to 10 μm, PM_10_) and fine particles (size less than or equal to 2.5 μm, PM_2.5_). Furthermore, particles whose size is smaller than 0.1 μm are called ultrafine particles [1,2].

The reason why it is important to study particulate matter is because it affects human health. In short, it affects lungs, harms the respiratory system, and reduces life expectancy [3]. According to [4], PM_2.5_ causes respiratory inflammation, cancer, and asthma [5,6,7]. Additionally, in accordance with [8], it affects both the cardiovascular system [9,10] and the nervous system [11], among others.

Urban parks are very good filters of particulate matter [12,13,14]. In accordance with [15,16,17], urban green spaces purify the air and improve the air quality. The report presented in [18] shows that trees and natural spaces contribute to reducing global warming and air pollution. In addition, [18] recommends increasing the amount of green spaces and the quality and connectivity between them. In [18], it is also recommended that one improve land-use planning policies, which is consistent with the conclusions of [13].

The above statement justifies the need for air pollution measurement systems that, on the one hand, can be deployed in large urban areas or in areas of interest [19,20,21,22,23], and that on the other hand can perform robust measurements of air pollution variables under study [13].

This article is a complement and continuation of the research works presented in [12,13,14]. In these papers, it was found that the data on air pollution collected inside the La Carolina Park, Quito, Ecuador, and on streets around that park, did not come from the same statistical population. That is, the fluctuations in the measurements were not only due to chance but were also significantly different from each other.

In order to make the comparison presented in [12,13,14], both parametric and non-parametric statistical inferences were used, confidence intervals were obtained, and hypothesis tests were performed based on the Wilcoxon signed-rank test, the Kruskal-Wallis test, and the Friedman’s test, among others. In addition, once the statistical evidence that the considered variables did not have the same distribution was established, the similarities and differences between these variables were determined based on a robust location and scale measurements. Therefore, the characteristics that differentiate the considered variables were established.

The use of robust statistics in [13] was due to the existence of outliers in the observations of all the variables, in the sense that these were observations that were very far from most of the data. A task that was pending in [12,13,14] was the establishment of robust intervals for the location measurements. Therefore, the main aim of this article is to solve this problem. Here, the comparison of the confidence intervals found by robust methods [24,25,26] with the confidence intervals found with non-parametric methods [27,28] is carried out. Comparisons are also made with classic confidence intervals and confidence intervals based on the bootstrap method [26].

In accordance with the aims and scope of this journal, this article is dedicated to performing the statistical analysis of the information from sensor measurements.

This article is structured as follows: Section 2 shows the study area and considered data, Section 3 is aimed at solving the problem of designing robust confidence intervals and showing the experimental results, and Section 4 is devoted to the conclusions of the research work.

## 2. Study Area and Considered Data

**Figure 1:**
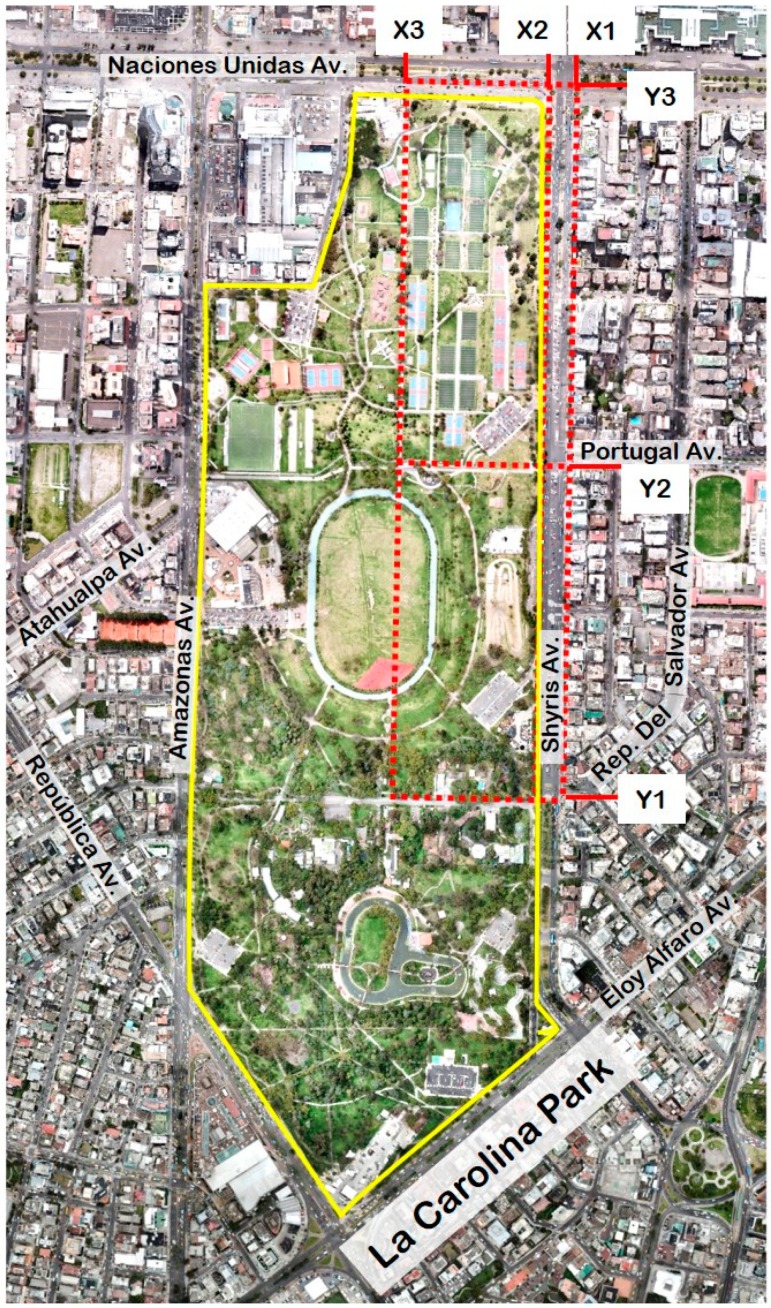
Park and route that was followed to perform the measurements. Urban park: Space delimited by the yellow lines; Route: Red dashed lines. (This figure has been taken from [13]).

As the study area is the same as in [12,13,14], this article shows only the figure that describes the park and the random variables considered. All this information has been taken directly from [13]. In accordance with [13], Figure 1 shows the park and routes that were followed to perform the measurements. The exact description of the random variables $X_{1}$, $X_{2}$, $X_{3}$, $Y_{1}$, $Y_{2}$, and $Y_{3}$ is given in [13]. Therefore, a simple explanation of these variables is given below.

- $X_{1}$ is a route that represents the sidewalk on Avenida de los Shyris (shown as Shyris Av. in Figure 1) that is in front of La Carolina Park.
- $X_{2}$ is a route that represents the sidewalk that is situated between La Carolina Park and Avenida de los Shyris.
- $X_{3}$ is a route through the center of the park.
- $Y_{1}$ is a route through Avenida República del Salvador (shown as Rep. Del Salvador Av. in Figure 1) that people follow to go to the center of the park.
- $Y_{2}$ is a route through Avenida Portugal (shown as Portugal Av. in Figure 1) that people use to go to the center of the park.
- $Y_{3}$ is a route that represents the sidewalk that is situated between La Carolina Park and Avenida Naciones Unidas (shown as Naciones Unidas Av. in Figure 1).

In accordance with [12,13,14], the PM_2.5_ measurement instrument used in this research was a portable CEL-712 Microdust Pro monitor paired with a GPS (Global Positioning System). The calibration results of the measurement instrument are shown in [13]. The measurements were performed at a walking speed of 2 km/h and a 1.5 m height, and from 8:00 a.m. to 10:00 a.m. because air pollution is the worst during these hours [13].

The conclusions of the analysis of the pollution levels obtained in [12,13,14] were that there are significant differences between the six variables analyzed and that the park acts as an air pollution filter. In [13], a statistical summary of each variable and graphics was initially made as a time series and box-plot. Thus, the number of observations available were presented, and some of the variables indicated more fluctuations than others. In addition, in almost all variables, the existence of extremely remote values on the right of the central group was observed, as well as a lack of normality in the distribution of the variables, which was due to the lack of symmetry. High values of the shape measures (skewness and kurtosis) were also observed in the variables.

In addition, in [13], a smoothing technique based on simple moving averages was used, with the aim of reducing the influence of each individual data [29]. Furthermore, in order not to suppress any observation, variable changes were made with a view to achieving the adjustment of the data. However, although it was possible to adjust the values of certain variables to heavy-tailed distributions, it was not possible to properly adjust all of the variables.

After having performed what was mentioned in the previous paragraphs, in [13], non-parametric bilateral confidence intervals were constructed, based on the Wilcoxon-Mann-Whitney test, in order to test whether the samples taken from the six variables came from a population that have a common median [27,28]. Thus, it was concluded that the variables were classified, according to the categories of the air quality index of Quito [30], into four groups: a group formed by variable $Y_{3}$, another group formed only by $Y_{2}$, a third group formed by variables $X_{1}$ and $X_{2}$, and, finally, the group formed by variables $X_{3}$ and $Y_{1}$.

Due to the high number of outliers in the variables, it was decided to use a robust analysis in [13]. Therefore, in order to find estimates where the center of symmetry of the distribution could be found, *L*-estimators of the location were used, which are linear combinations of order statistics [24,25].

On the other hand, to determine the variability of the data, different scale estimators were used. Specifically, the mean of the deviations from the mean (${\mathrm{MAD}}_{\mathrm{mean}}$), the median absolute deviation (MAD), and the semi interquartile range (SIR) were used. Additionally, the biweight midvariance scale estimator ($S_{bi}\left(c\right)$) was used, which is based on an *M*-estimator of the location, since it has a greater efficiency than conventional scale measurements. Finally, the least median of squares (LMS) punctual estimator was used [31,32].

Robust statistics are characterized by the influence curve, which shows the influence that an observation can have compared to the rest of the observations [31]. In the case of non-robust estimators, these influence curves are not bounded. Therefore, the appearance of an observation that is considerably far from most of the data greatly affects non-bounded curves of influence and, therefore, non-robust estimators. But they do not have such a strong influence on robust estimates. Therefore, in [13], it was determined that the MAD, SIR, and LMS estimates were the most stable, and the variables were classified based on the scale estimators. These influence curves also have other properties that differentiate them from each other, such as being continuous or differentiable.

## 3. Robust Confidence Intervals: Results

### 3.1. Method

A standard way to establish confidence intervals, as well as hypothesis contrasts, is to consider that the statistic given by Equation (1) follows a Student’s *t*-distribution with n−1 degrees of freedom:

(1) $$T=\frac{\sqrt{n}\left(\bar{x}-\mu \right)}{s}$$

where n is the sample size, $\bar{x}$ is the sample mean, μ is the expected mean value, and s is the sample standard deviation. Thus, the classic confidence intervals for the mean are of the form:

(2) $$\left(\bar{x}\pm t_{n-1,\alpha /2}\times \frac{s}{\sqrt{n}}\right)$$

where α is the significance level and 1−α is the confidence level. In this way, the distribution of T is symmetric and zero mean. These hypotheses are usually true if the distribution from which the sample was obtained is approximately Gaussian. However, in the case under study the hypotheses mentioned above are not met, because the variables are heavy tails. As indicated in [24], these deviations from the starting assumptions have as a basic problem the increase in the length of the confidence intervals, since increasing the standard deviation increases that length. In addition, there are other problems related to hypothesis contrasts, such as controlling the probability of a type I error (that is, rejecting the null hypothesis when it is true), because the estimator that is used is biased [26].

For the moment, confidence intervals of the form given by Equation (3) will be established, based on analyses performed in [24]:

(3) $$T\pm \frac{t^{*}\times \omega }{\sqrt{n}}$$

where T is a location estimator, ω is a scale estimator, $t^{*}$ is a constant related to the Student’s *t*-distribution, and n is the sample size. For robust intervals, estimators based on point statistics and three families of estimators will be considered.

In this sense, it is important to mention that the a-trimmed mean family, with 0≤a≤0.5 [24,25], consists in suppressing a×100% observations from both the left and the right, and then finding the average of the observations not suppressed. For this estimator, an approximation of its standard deviation is the Winsorized standard error of order a of the sample $\left(X_{1},\;\ldots ,\;X_{n}\right)$, $s^{W}(a)$ [26].

In a few words, if the ordered sample is $X_{\left(1\right)}\leq X_{\left(2\right)}\leq \ldots \leq X_{\left(n\right)}$ and k=⎣n·a⎦, with ⎣h⎦ being the floor function of the positive real number h, then the Winsorized sample, $W_{\left(1\right)}\leq W_{\left(2\right)}\leq \ldots \leq W_{\left(n\right)}$, is obtained by changing the k lowest values of the sample with $X_{\left(k+1\right)}$ and the k highest values of the sample with $X_{\left(n-k\right)}$. In this article, the family of estimators based on the Andrew’s wave and the family based on biweight estimators are also used [26]. Therefore, the following pairs of estimators will be considered:

1. Mean and standard deviation, $\left(\bar{x},s\right)$ [13].
2. Median and median absolute deviation, (M, MAD) [13].
3. Median and semi interquartile range, (M, SIR) [13].
4. a-trimmed mean [13] and Winsorized standard error of order a, $\left(T\left(a\right),\;s^{W}\left(a\right)\right)$ [26]:
  (4) $$T\left(a\right)=\frac{1}{n-2\left[n\cdot a\right]}\overset{n-\left[n\cdot a\right]}{\underset{i=\left[n\cdot a\right]+1}{\sum }}X_{\left(i\right)}$$
  (5) $$s^{W}\left(a\right)=\sqrt{\frac{1}{n-1}\overset{n}{\underset{i=1}{\sum }}{\left(W_{i}-{\bar{x}}_{a}^{W}\right)}^{2}}$$
  where ${\bar{x}}_{a}^{W}$ is the mean of the a-Winsorized sample.
5. Andrew’s wave, $\left(T_{\omega a},\;s_{\omega a}\right)$ [26]:
  If
  (6) $$u_{i}=\frac{x_{i}-M}{c\cdot MAD},\;c=2.4\cdot \pi$$
  Then
  (7) $$T_{\omega a}=M+c\cdot MAD\cdot arctan\left(\frac{\sum_{\left|u_{i}\right|<1}sen\left(\pi \cdot u_{i}\right)}{\pi \cdot \sum_{\left|u_{i}\right|<1}cos\left(\pi \cdot u_{i}\right)}\right)$$
  (8) $$s_{\omega a}=c\cdot MAD\cdot \frac{\sqrt{n\cdot \sum_{\left|u_{i}\right|<1}sin^{2}\left(\pi \cdot u_{i}\right)}}{\pi \cdot \left|\sum_{\left|u_{i}\right|<1}cos\left(\pi \cdot u_{i}\right)\right|}$$
6. Biweight, $\left(T_{bi},\;s_{bi}\right)$ [13]:

If

(9) $$u_{i}=\frac{x_{i}-M}{c\cdot MAD},\;c=9$$

then

(10) $$T_{bi}=M+\frac{\sum_{\left|u_{i}\right|<1}\left(x_{i}-M\right){\left(1-u_{i}^{2}\right)}^{2}}{\sum_{\left|u_{i}\right|<1}{\left(1-u_{i}^{2}\right)}^{2}}$$

(11) $$S_{bi}=\frac{\sqrt{n\cdot \sum_{i=1}^{n}{\left(x_{i}-M\right)}^{2}{\left(1-u_{i}^{2}\right)}^{4}}}{\left|\sum_{i=1}^{n}\left(1-u_{i}^{2}\right)\left(1-5\cdot u_{i}^{2}\right)\right|}$$

### 3.2. Confidence Interval for Each Parameter

Table 1 shows the value of the estimators for each variable. However, in accordance with [24], taking into account situations with Gaussian distributions, with an outlier (one-wild), or with trimmed distributions (slash), the best results in terms of efficiency are obtained with the M-estimators of the location, that is, with the last two pairs of estimators of Table 1.

Once the estimators were selected, the next step was to establish the $t^{*}$ constants of Equation (3). In order to do this, according to [33,34], for the first pair of robust estimators (M, MAD), the percentiles of a Student’s t-distribution with n−2 degrees of freedom were chosen. For the second pair of robust estimators (M, SIR), $t^{*}=t_{n-1}/1.075$ was chosen. The constant $t^{*}$ for the family $\left(T\left(a\right),\;s^{W}\left(a\right)\right)$ was taken from the Student’s t-distribution with n−2×n×a−1 degrees of freedom. For the families of M-estimators based on the Andrew’s wave and on the biweight estimators, the percentiles of a Student’s t-distribution with 0.7×(n−1) degrees of freedom were chosen. In the case where 0.7×(n−1) was not an integer, the next integer greater than 0.7×(n−1) was chosen as the degree of freedom of the Student’s t-distribution.

In addition to the previous confidence intervals, another interval was included for the median that was performed using the bootstrap-t method [26]. With *a* (1−α) confidence level, this confidence interval was given by Equation (12):

(12) $$\left(M-t_{1-\alpha /2}^{*}\cdot s^{*},\;M+t_{\alpha /2}^{*}\cdot s^{*}\right)$$

where M is the median of the original sample, and, for the bth bootstrap sample, b=1, …, B, $s^{*}$ is the unbiased estimator of the standard deviation, $M^{*}$ is the median, and $t_{1-\alpha /2}^{*}$ and $t_{\alpha /2}^{*}$ are the percentiles of the statistic $M_{b}^{*}$ given by Equation (13):

(13) $$M_{b}^{*}=\frac{M^{*}-M}{s^{*}}$$

For the case under study, as the number of samples for each of the variables was greater than 70, B=499 bootstrap samples were generated in order to ensure that (1−α)=0.95 was proportional to 1/(B+1).

Table 2 shows the length of the confidence interval (see Equation (3)) of each pair of estimators. Additionally, both the intervals obtained by using the bootstrap-t method and the nonparametric intervals obtained in [13] are shown in Table 2.

**Figure 2:**
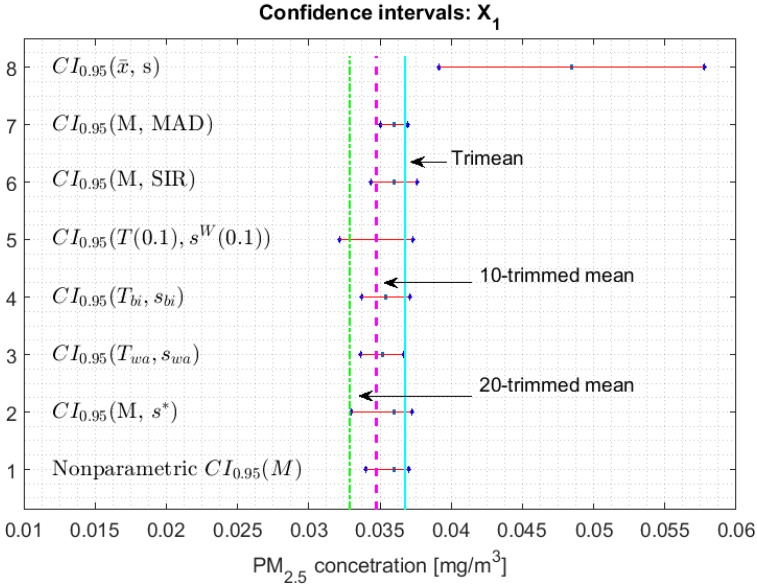
95% confidence intervals $\left(CI_{0.95}\right)$ for robust and non-robust estimators: $X_{1}$.

**Figure 3:**
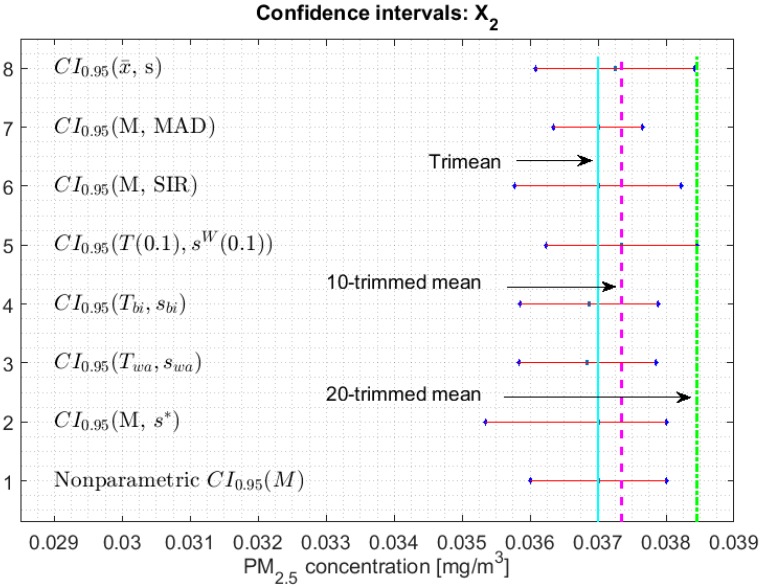
95% confidence intervals $\left(CI_{0.95}\right)$ for robust and non-robust estimators: $X_{2}$.

**Figure 4:**
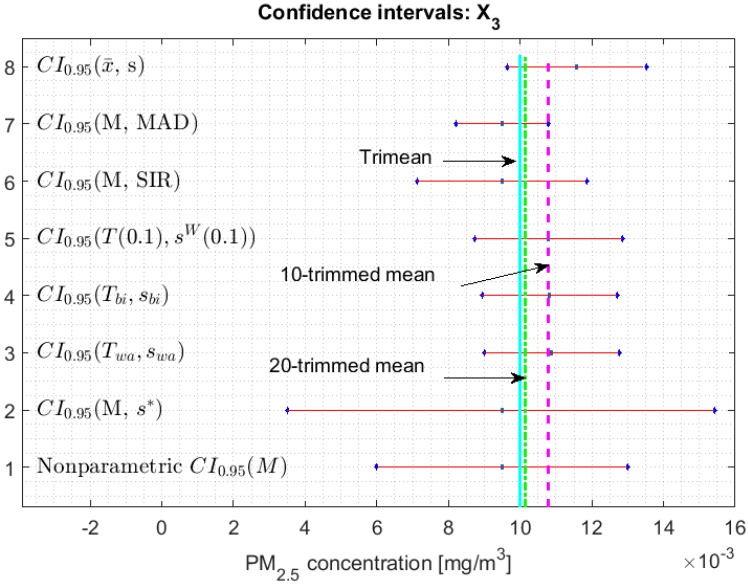
95% confidence intervals $\left(CI_{0.95}\right)$ for robust and non-robust estimators: $X_{3}$.

**Figure 5:**
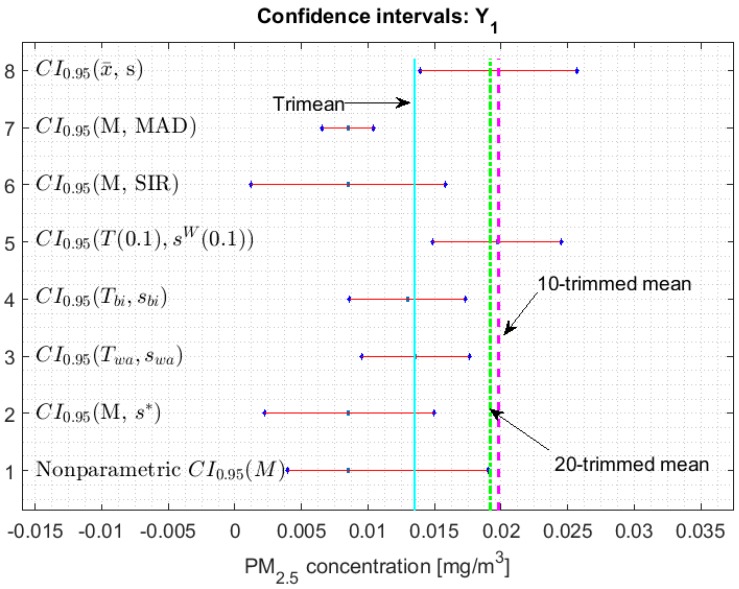
95% confidence intervals $\left(CI_{0.95}\right)$ for robust and non-robust estimators: $Y_{1}$.

**Figure 6:**
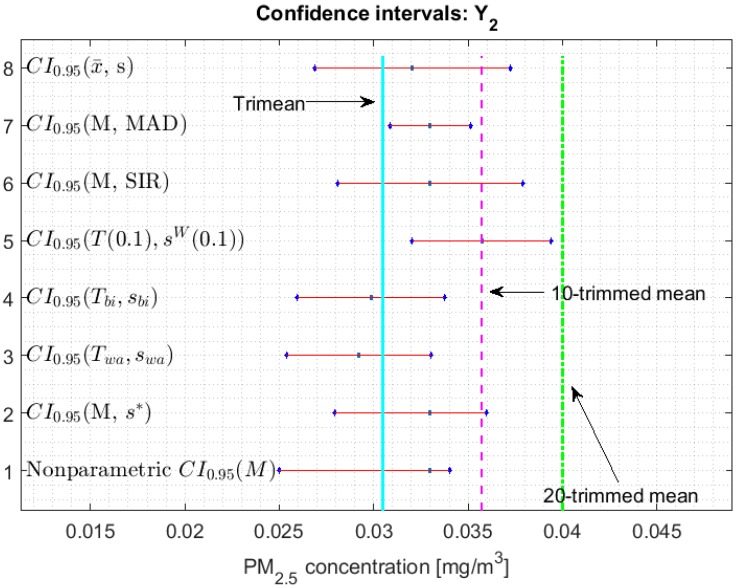
95% confidence intervals $\left(CI_{0.95}\right)$ for robust and non-robust estimators: $Y_{2}$.

**Figure 7:**
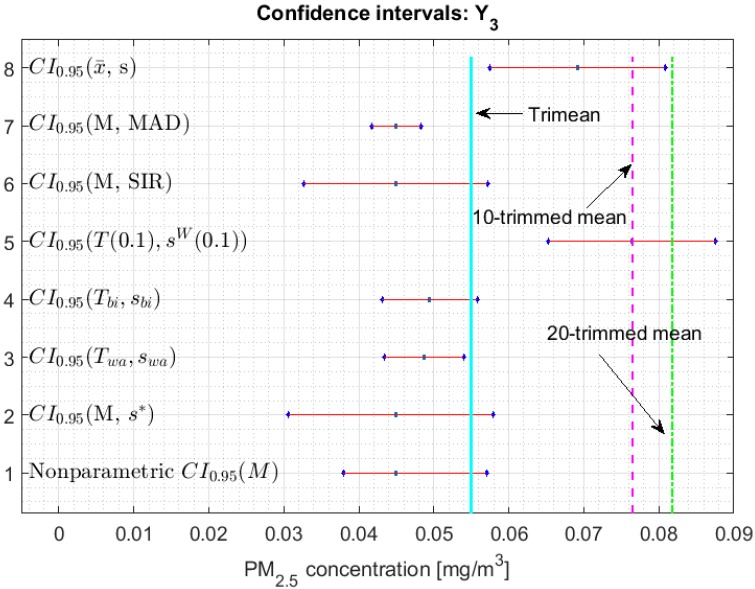
95% confidence intervals $\left(CI_{0.95}\right)$ for robust and non-robust estimators: $Y_{3}$.

Figure 2, Figure 3, Figure 4, Figure 5, Figure 6 and Figure 7 show the confidence intervals that have been built, together with the trimean [13] and the a-trimmed mean (Equations (4) and (5)) for 10% and 20% pruning on each side of the samples. In these figures, it can be seen that the confidence intervals based on the Andrew’s wave and the biweight estimators are practically the same for all variables. The intervals built from the median and the median absolute deviations are the smallest in all the variables, and for most of the variables they neither contain the trimean nor the a-trimmed mean. The nonparametric intervals for the median contain the robust intervals based on the Andrew’s wave and those based on the biweight estimators, being generally a little wider. In addition, the nonparametric intervals contain almost all other intervals built with robust estimators, except those based on the a-trimmed mean and Winsorized standard error of order a.

In addition, the fact that in some variables the intervals built with the family of estimators based on the a-trimmed mean, for a=0.1, appear somewhat longer than others and slightly shifted to the right, indicates that they are more influenced by observations away from the center of the data on the right. The trimean of each variable is found in all non-classical intervals, except for those based on the median and the median absolute deviation. However, the a-trimmed mean with both 10% and 20% are not always found in the built confidence intervals.

In accordance with [13], $X_{1}$ has a bias towards the right. In addition, it is the only variable that has values that exceed all levels of air quality, which is why the confidence interval based on the mean and the standard deviation is very wide (see Figure 2). This interval is shifted towards high values and does not even cover the value of the median or the other robust location estimators. That is, it is greatly influenced by these extreme observations.

With respect to $X_{2}$, in [13] it was shown that this variable is considered among the variables that exhibit a better behavior, taking into account both the location and dispersion measures. Therefore, this variable can be considered to be light tails. For this reason, all the confidence intervals shown in Figure 3 are very similar and are also smaller, in each type of interval, than the intervals of the rest of the variables. Like $X_{1}$, $X_{2}$ only contains the trimean and a-trimmed mean for 10% pruning, while the a-trimmed mean for 20% pruning is only contained in the classical interval and in the interval built by using the a-trimmed mean and Winsorized standard error of order a, for a=0.1.

Although, in [13], it was shown that $X_{3}$ is the variable with many observations with very low values, it also has a distribution with light tails and has more variability than $X_{2}$. For this reason, the nonparametric confidence interval (see Figure 4) is almost twice as wide as the other confidence intervals, except in the case of the interval found by the bootstrap method. In addition, it is important to mention that all intervals practically contain the considered location measurements. The interval constructed using the bootstrap method is among those with the longest length of all the variables, except for the confidence intervals of the $Y_{3}$ variable (see Figure 7).

It has already been established in [13] that among the variables with a greater variability there is $Y_{1}$, which has few extreme observations and a bias towards high values, and it can be seen that the lengths of the confidence intervals are also large but are displaced towards low values of the variable (see Figure 5). This is because, by suppressing the extreme observations, the remaining observations are concentrated in low values of the variable. The above justifies the fact that the interval based on the a-trimmed mean and Winsorized standard error of order a, for a=0.1, does not contain the trimean. Finally, taking into account the similarity of the confidence intervals and the fact that the a-trimmed means for 10% pruning are outside the nonparametric confidence interval, it is observed that the a-trimmed means are only contained in the classical confidence interval and in the interval that is centered on T(0.1).

With respect to $Y_{2}$, in [13] it was determined that it does not resemble the rest of the variables, as far as centralization measures are concerned. This variable has many observations that influence the variability. In addition, together with $X_{2}$ and $X_{3}$, $Y_{2}$ is the variable in which the confidence intervals are more similar to each other (see Figure 6). In the case of $Y_{2}$, it is observed that no confidence interval contains the a-trimmed mean for 20% pruning.

Having determined in [13] that the distribution of the values of $Y_{3}$ may correspond to a distribution of heavy tails, with bias towards high values, it is already possible to corroborate that the confidence interval based on the fact that the mean and standard deviation are offset from the remaining intervals (see Figure 7), except in the case of the interval based on the a-trimmed mean and Winsorized standard error of order a, for a=0.1, which only suppresses 10% of the observations at each end. Furthermore, according to [13], $Y_{3}$ is the variable with more observations that exceed the acceptable level of the PM_2.5_ concentration [30] and the one with the greatest variability. This is also ratified, because firstly their confidence intervals are the most extensive among all the variables, and secondly only the confidence intervals found through bootstrap techniques, nonparametric intervals, and some robust intervals contain the trimean.

**Figure 8:**
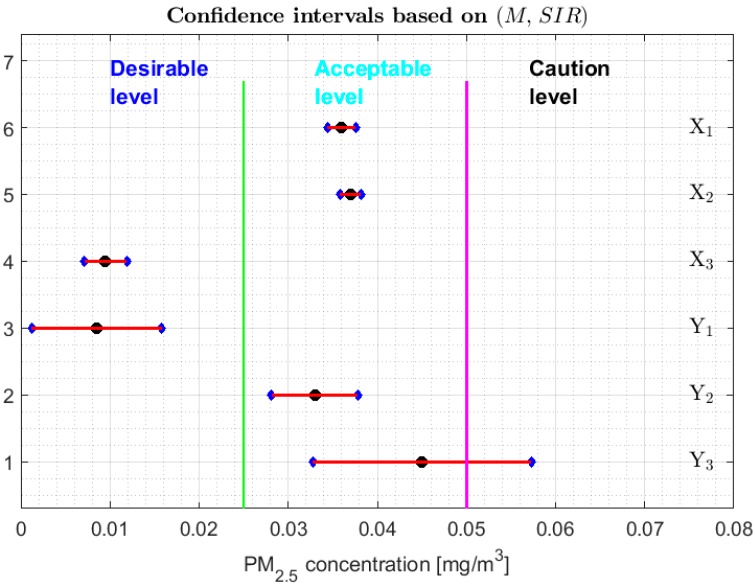
95% confidence intervals based on (M, SIR), and the bands that delimit the three lowest categories of air pollution by PM_2.5_ concentrations in Quito. Median: Black circle; Ends of the intervals: Blue diamonds.

**Figure 9:**
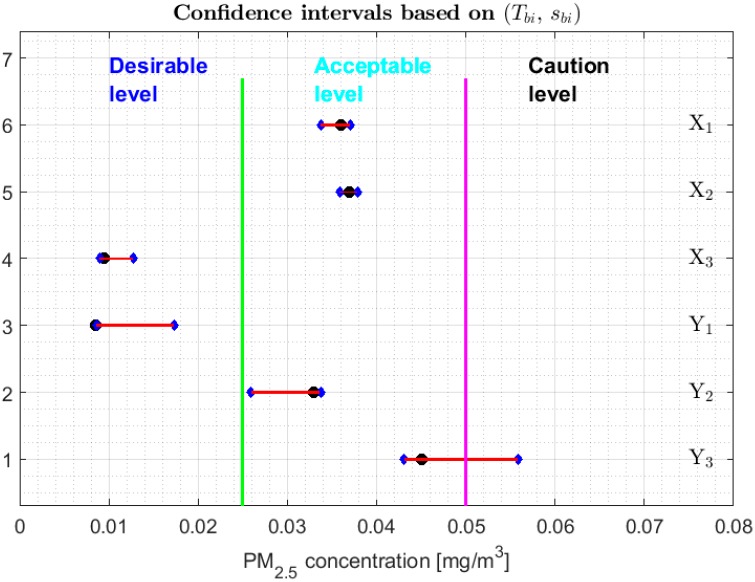
95% confidence intervals based on $\left(T_{bi},\;s_{bi}\right)$, and the bands that delimit the three lowest categories of air pollution by PM_2.5_ concentrations in Quito. Median: Black circle; Ends of the intervals: Blue diamonds.

**Figure 10:**
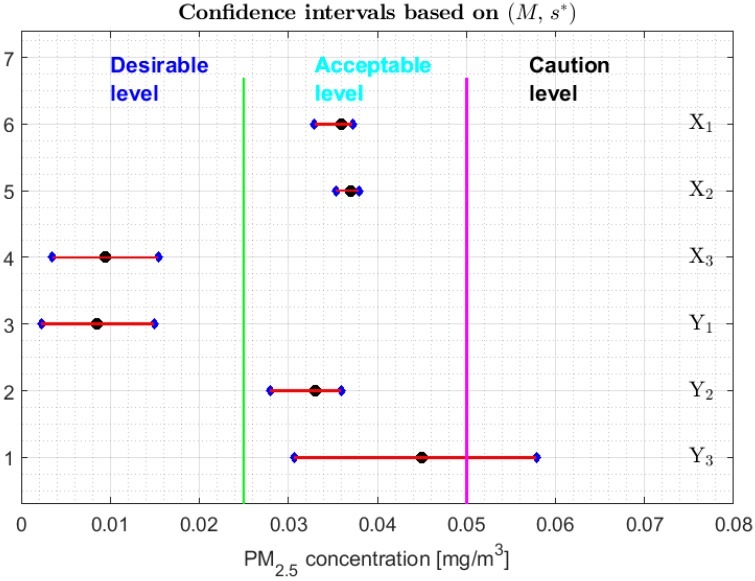
95% confidence intervals based on $\left(M,s^{*}\right)$, and the bands that delimit the three lowest categories of air pollution by PM_2.5_ concentrations in Quito. Median: Black circle; Ends of the intervals: Blue diamonds.

As was done in [13], Figure 8, Figure 9 and Figure 10 show 95% confidence intervals for the medians of the variables under study and different categories of air pollution by PM_2.5_ that are defined in [29]. However, unlike [13], the robust confidence intervals constructed by using the following pairs are presented here: (M, SIR), $\left(T_{bi},\;s_{bi}\right)$, and $\left(M,s^{*}\right)$. It is important to mention that the confidence intervals based on the Andrew’s wave have not been included, firstly due to the analogy of these with the intervals based on the biweight statistic and, secondly, because the intervals based on the biweight statistic are shorter than those based on the Andrew’s wave. The confidence intervals based on (M, MAD) have not been included because they are a subset of the confidence intervals based on (M, SIR). In addition, the confidence intervals based on the 0.1-trimmed mean have also not been included, because these are not built for the median but for T(0.1). On the other hand, the bands that delimit the three lowest categories in which the air quality is classified in the city of Quito have been included, according to the levels of air pollution in this city by PM_2.5_ concentrations [30].

From Figure 8, Figure 9 and Figure 10, it can be seen that for the confidence intervals found with the biweight estimators (see Figure 9) it is possible to discriminate more precisely the equality of the medians, obtaining results comparable to those obtained with the non-parametric intervals. That is, the variables can be classified into the following four groups: $\left\{Y_{2}\right\}$, $\left\{Y_{3}\right\}$, $\left\{X_{1},\;X_{2}\right\}$, and $\left\{X_{3},\;Y_{1}\right\}$. In addition, the medians of $X_{3}$ and $Y_{1}$ are strictly contained in the Desirable level, the medians of the variables $X_{1}$, $X_{2}$ and $Y_{2}$ are contained in the Acceptable level, and it is rejected that these medians may belong to the other levels. Furthermore, the median of variable *Y*_3_ can be in the Acceptable level or Caution level.

With respect to the families of the confidence intervals based on (M, SIR) and $\left(M,s^{*}\right)$, shown respectively in Figure 8 and Figure 10, the following can be said:

1. One cannot reject that variables $X_{1}$ and $X_{2}$ can have equal medians, and, with a 95% confidence level, it is rejected that they are the medians of any of the other variables, since the acceptance limits of the first two variables do not include the acceptance limits of the others.
2. One cannot reject that the variables $X_{3}$, $Y_{1}$, $Y_{2}$, and $Y_{3}$ can have equal medians.
3. When removing the observations of the tails of the distributions, the variables $X_{1}$ and $X_{2}$ are those that present less variability, and the variables $Y_{1}$ and $Y_{3}$ have more variability than the rest.

At this point, both [13] and this article have shown that the variables that have the greatest variability are $X_{1}$ and $Y_{3}$. These variables have the worst behavior, with very high values, because they contain critical pollution points. However, as the other variables are either routes followed through the center of the park or at the edges of the park, the pollution levels on these routes are not as critical. Therefore, the section will be finalized by showing the robust confidence band graphs for variables $X_{1}$ and $Y_{3}$, because these are the variables that have more observations shifted towards high values. Representing the graphs of the robust confidence bands for the other variables, which have a good behavior, does not contribute significantly to this article from a scientific point of view.

### 3.3. Robust Confidence Band Graphs

**Figure 11:**
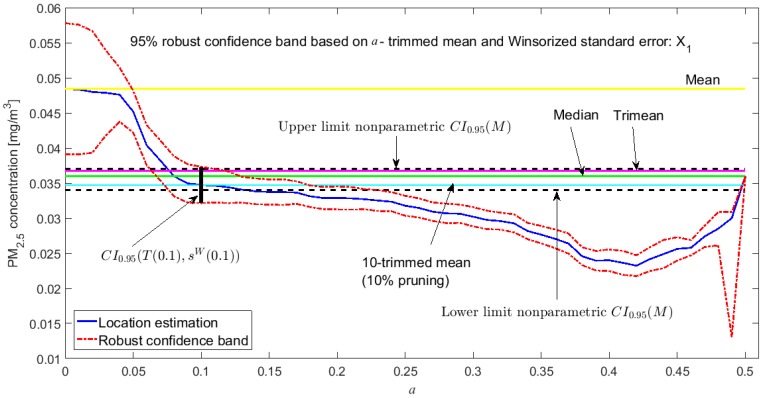
95% robust confidence band based on the a-trimmed mean and Winsorized standard error: $X_{1}$.

**Figure 12:**
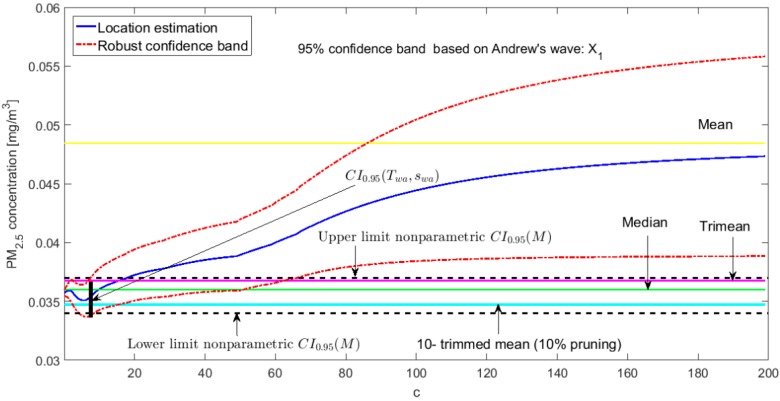
95% robust confidence band based on the Andrew’s wave: $X_{1}$.

**Figure 13:**
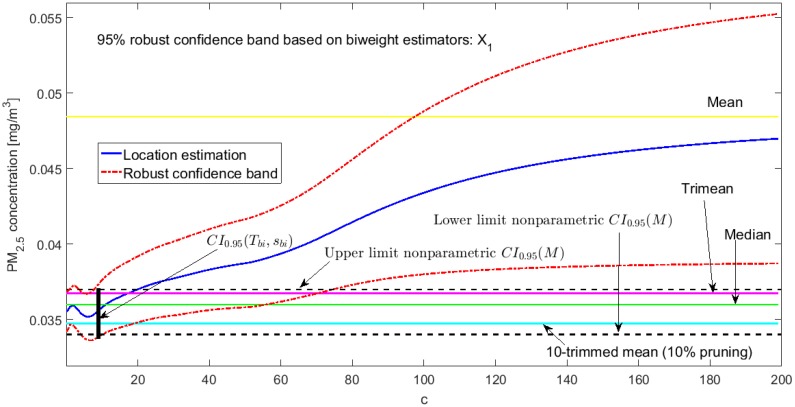
95% robust confidence band based on the biweight estimators: $X_{1}$.

**Figure 14:**
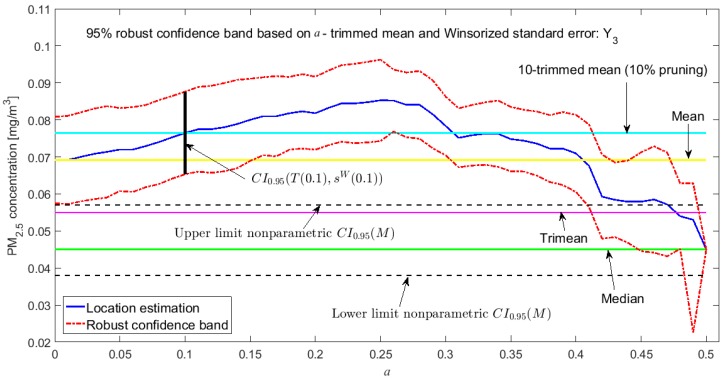
95% robust confidence band based on the a-trimmed mean and Winsorized standard error: $Y_{3}$.

**Figure 15:**
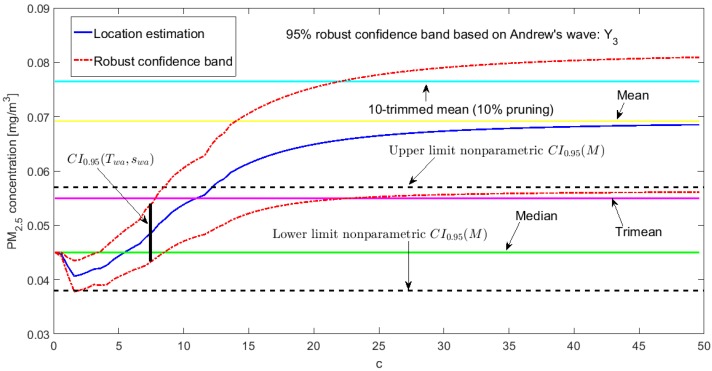
95% robust confidence band based on the Andrew’s wave: $Y_{3}$.

**Figure 16:**
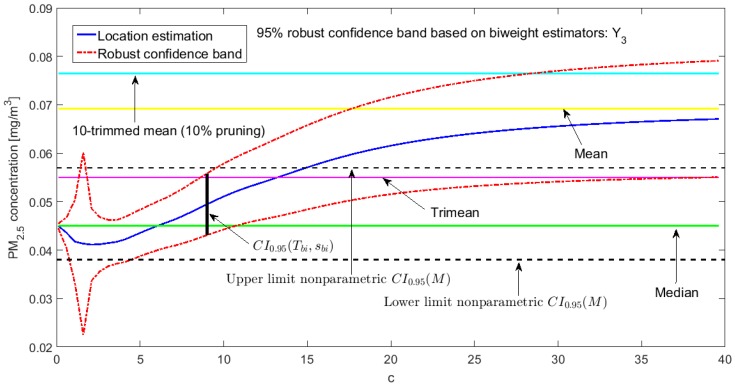
95% robust confidence band based on the biweight estimators: $Y_{3}$.

The graphs of the robust confidence bands for $X_{1}$ and $Y_{3}$ are shown in Figure 11, Figure 12, Figure 13, Figure 14, Figure 15 and Figure 16. The families of estimators based on the a-trimmed means, Andrew’s wave, and biweight estimators have been taken into account, because their location estimators’s influence curves are bounded and are softer than the influence curves of the rest of the location estimators [24].

From Figure 11, Figure 12, Figure 13, Figure 14, Figure 15 and Figure 16, for the estimators $\left(T\left(a\right),\;s^{W}\left(a\right)\right)$, it is observed that for $X_{1}$ there is a slight decrease in the location measurements and a constant amplitude of the confidence band (see Figure 11). In the graph corresponding to $Y_{3}$ (see Figure 14), the location measurements change their trend, but the increase in the amplitude of the confidence band is much more noticeable. Moreover, in this graph the confidence band does not contain either the median or the trimean, unless only 10% of the central observations are taken into account.

For the estimators $\left(T_{\omega a},\;s_{\omega a}\right)$, it is observed that for low values of c the estimates are hardly affected by anomalous observations (see Figure 12 and Figure 15). However, as c increases, the center of the interval increases, as does its amplitude, because they contemplate observations in a greater range. However, the confidence band for $Y_{3}$ (see Figure 15) is much wider than for $X_{1}$ (see Figure 12). This also happens for the family of estimators $\left(T_{bi},\;s_{bi}\right)$ (see Figure 13 and Figure 16), although it is true that the amplitude of the band for $X_{1}$ increases proportionally more than for $Y_{3}$.

For the estimators $\left(T_{bi},\;s_{bi}\right)$, as in the previous case for low values of c, only observations close to the median are contemplated, and, as c increases, wider confidence intervals are obtained. For low values of c, the estimator $s_{bi}$ shows many fluctuations. Therefore, the confidence band is much more variable. Furthermore, the confidence intervals chosen for $X_{1}$ (see Figure 13) and $Y_{3}$ (see Figure 16) contain the median and the trimean.

Finally, it is important to note that $Y_{3}$ has much more variability than $X_{1}$, although the latter has higher observations than the rest of the variables. It is also confirmed that the confidence intervals for $Y_{3}$ are always wider. The behavior of the families of the estimators $\left(T_{\omega a},\;s_{\omega a}\right)$ and $\left(T_{bi},\;s_{bi}\right)$ is very similar, and the intervals they generate are narrower than the nonparametric intervals that were found. The behavior of the family $\left(T\left(a\right),\;s^{W}\left(a\right)\right)$ is very different from that of the other two families. This family produces confidence intervals with a more constant amplitude, but it cannot be assured that these intervals contain the median. In addition, there is a large difference between the confidence intervals for $X_{1}$ and $Y_{3}$.

## 4. Conclusions

In this article, a response has been given to a problem that was pending to be solved in previous research articles [13]. Specifically, robust confidence intervals were found for PM_2.5_ concentration measurements in the urban park called La Carolina, Quito, Ecuador. The main contributions of this article were that different techniques were applied for the construction of robust confidence intervals, that their results were compared, and that the results of the design of these robust intervals were applied to analyze whether the six variables considered in the study came from the same distribution, establishing the differences between the parameters that characterized those variables.

For the construction of the confidence intervals, the classical, non-parametric, bootstrap, and, mainly, several pairs of robust statistics were used. Classic confidence intervals make use of the hypothesis that the observations come from a Gaussian, or approximately Gaussian, distribution. However, what happened was that the variables that were given contained numerous observations with extreme values on the right, which did not meet the hypothesis that was assumed.

From the analysis of the variables under study, the following was concluded: (1) the median of $Y_{3}$ was greater than that of all other variables; (2) the median of $Y_{2}$ was different from all the medians of the other variables; (3) the medians of $X_{1}$ and $X_{2}$ could be the same but different from all others (that is, lower than the median of $Y_{3}$ but greater than the median of the other variables); and (4) the same as (3) happened with the medians of $X_{3}$ and $Y_{1}$, which could be the same and smaller than the others.

Speaking in terms of air pollution and urban planning, the variable that could concern the citizen who lives and/or works around the La Carolina park is $Y_{3}$ because in [13] and in this article it has been shown that this variable presents location estimates that are remarkably higher than the rest of the variables, these estimates being between the Acceptable and the Caution levels. In addition, this variable is the one that provides higher scale estimates, showing differences with the remaining behavior patterns. The foregoing observation is in accordance with the location of the street on which the route to be followed to measure $Y_{3}$ was drawn. In [12,13,14], it was shown that both the direction in which the wind blows and the type of circulation around La Carolina Park render the Avenida Naciones Unidas (shown as Naciones Unidas Av. in Figure 1), which has been represented by $Y_{3}$, the most likely to have higher levels of air pollution due to PM_2.5_ concentrations. Therefore, the conclusions given by the authors of [12,13,14] are ratified.

Before finalizing the conclusions of this article, it is important to emphasize the meaning of the results that have been obtained in this research work. Therefore, it is important to highlight that the statistical analysis presented in this article has been conceptual and that this analysis has focused on summarizing a set of data into a few values that are representative of that set. In this way, it has been possible to characterize the population under study using these few representative values of the above-mentioned set. In addition, what has been said above has been done using robust techniques, which do not take into account all the values that have been collected in the sample of the population of interest. Specifically, the use of robust techniques has allowed extreme values to have little influence on the process of characterization of the sample of the population of interest.

## Figures and Tables

**Table 1:** The robust location and scale estimators.

Variable	$\bar{x}$	s	M	MAD	M	SIR
	$\left(mg/m^{3}\right)$	$\left(mg/m^{3}\right)$	$\left(mg/m^{3}\right)$	$\left(mg/m^{3}\right)$	$\left(mg/m^{3}\right)$	$\left(mg/m^{3}\right)$
$X_{1}$	0.0485	0.0589	0.0360	0.0060	0.0360	0.0110
$X_{2}$	0.0373	0.0054	0.0370	0.0030	0.0370	0.0060
$X_{3}$	0.0116	0.0099	0.0095	0.0065	0.0095	0.0130
$Y_{1}$	0.0198	0.0247	0.0085	0.0080	0.0085	0.0330
$Y_{2}$	0.0321	0.0218	0.0330	0.0090	0.0330	0.0220
$Y_{3}$	0.0692	0.0531	0.0450	0.0150	0.0450	0.0600
**Variable**	T(0.1)	$s^{W}\left(0.1\right)$	$T_{\omega a}$	$s_{\omega a}$	$T_{bi}$	$s_{bi}$
	$\left(mg/m^{3}\right)$	$\left(mg/m^{3}\right)$	$\left(mg/m^{3}\right)$	$\left(mg/m^{3}\right)$	$\left(mg/m^{3}\right)$	$\left(mg/m^{3}\right)$
$X_{1}$	0.0347	0.0130	0.0351	0.0096	0.0354	0.0106
$X_{2}$	0.0373	0.0041	0.0368	0.0046	0.0369	0.0046
$X_{3}$	0.0108	0.0083	0.0109	0.0095	0.0108	0.0096
$Y_{1}$	0.0197	0.0162	0.0136	0.0168	0.0130	0.0180
$Y_{2}$	0.0357	0.0122	0.0292	0.0160	0.0299	0.0162
$Y_{3}$	0.0765	0.0404	0.0487	0.0239	0.0495	0.0288

**Table 2:** The length of the confidence intervals at (1−α)=0.95.

Pair of Estimators	$\mathrm{Length}\;\mathrm{of}\;\mathrm{the}\;\mathrm{Confidence}\;\mathrm{Interval}\;\left(mg/m^{3}\right)$					
	$X_{1}$	$X_{2}$	$X_{3}$	$Y_{1}$	$Y_{2}$	$Y_{3}$
$\left(\bar{x},s\right)$	0.0186	0.0023	0.0039	0.0118	0.0104	0.0234
(M, MAD)	0.0019	0.0013	0.0026	0.0038	0.0043	0.0066
(M, SIR)	0.0032	0.0024	0.0048	0.0146	0.0098	0.0245
$\left(T\left(0.1\right),\;s^{W}\left(0.1\right)\right)$	0.0052	0.0022	0.0041	0.0097	0.0073	0.0223
$\left(T_{\omega a},\;s_{\omega a}\right)$	0.0030	0.0020	0.0038	0.0081	0.0077	0.0106
$\left(T_{bi},\;s_{bi}\right)$	0.0034	0.0020	0.0038	0.0087	0.0078	0.0127
$\left(M,s^{*}\right)$	0.0027	0.0027	0.0119	0.0127	0.0080	0.0272
Nonparametric [13]	0.0030	0.0020	0.0070	0.0150	0.0090	0.0190
